# Practical, high-speed Gaussian coherent state continuous variable quantum key distribution with real-time parameter monitoring, optimised slicing, and post-processed key distillation

**DOI:** 10.1038/s41598-023-47517-7

**Published:** 2023-12-06

**Authors:** Amanda Weerasinghe, Muataz Alhussein, Adam Alderton, Adrian Wonfor, Richard Penty

**Affiliations:** Electrical Engineering Division, Department of Engineering, University of Cambridge, Cambridge, CB3 0FA UK

**Keywords:** Electrical and electronic engineering, Fibre optics and optical communications, Quantum information, Quantum optics

## Abstract

Gaussian coherent state continuous variable quantum key distribution has gained interest owing to its security and compatibility with classical coherent optical fibre networks. For successful system deployment it is necessary to implement practical high speed systems which distil keys efficiently. Here, we demonstrate a Gaussian coherent state continuous variable quantum key distribution system at a 50 MHz symbol rate. Unlike most demonstrations to date which measure excess noise and infer key rates from this, we record signals in real time and distil keys. We also demonstrate, for the first time, slice reconciliation with optimised guard bands to maximise achievable secret key rates. Using this optimisation with multilevel slicing, a record 5 Mb/s secret key rate after a transmission distance of 25 km is achieved. This is a significant improvement on the 3 Mb/s secret key rate which is achieved with single level optimised slice reconciliation.

## Introduction

Quantum Key Distribution was originally proposed using discrete variables^1^, subsequently, the use of Gaussian modulated coherent states (GMCS) in continuous variable quantum key distribution has been proposed^2, 3^ and demonstrated^4, 5^. These cryptographic protocols, unlike current public key encryption standards, are resistant to code breaking by quantum computers^1, 2^. It is information theoretically secure, in contrast to the newly adopted post-quantum algorithms^6^, which rely on computational complexity and might be proven to have weaknesses in the future, CV-QKD is information theoretically secure, based on the quantum properties of coherent optical states.

CV-QKD systems can easily be integrated with classical coherent optical fibre networks since they are based upon classical coherent communication equipment. They have been proven to be secure against both collective eavesdropping attacks^7^ and also coherent attacks^8–10^, providing high security in practical implementation. Recent GMCS CV-QKD experiments have shown excess noise values which have enabled predictions of secret key rates up to a few Mb/s for metropolitan transmission distances^4, 11–15^. The GMCS CV-QKD system reported here has achieved a record 3.7 Mb/s secret key rate for a transmission distance of 30 km.

### Practical GMCS CV-QKD systems

Most existing CV-QKD demonstrations rely on bulky and expensive laboratory equipment such as arbitrary waveform generators and real time oscilloscopes to generate and record signals. In order to build practical CV-QKD systems that can be deployed in the field, it is crucial to investigate the use of commercially available, low-cost digital to analogue converters (DAC) and analogue to digital converters (ADC) for real-time data generation at sender and data recording at receiver respectively. In addition, most CV-QKD demonstrations to date estimate the secret key rates with measured parameters without real secret key distillation. Thus, it is also essential to demonstrate real-time parameter estimation, post processing, and secret key distillation to achieve fully operational, practical CV-QKD systems.

### Slice reconciliation for CV-QKD

Discrete variable (DV) QKD operates at a high rate of single photons per second. Although CV-QKD usually has a lower symbol rate, this can be overcome by encoding more than 1 bit per pulse, in practical systems.This results in high secret key rates for systems with appropriate signal to noise ratio. This is achieved by slice reconciliation that converts Gaussian continuous variables into binary strings of data^16, 17^. Conversion of continuous correlated data at Alice and Bob into binary data is a necessary step in CV-QKD, so that conventional binary error correction codes can be utilised for post processing to distil secret keys.

At metropolitan distances, CV-QKD achieves high secret key rates when received signal to noise ratio (SNR) is moderately high. However, even at moderately high received SNRs, CV-QKD systems exhibit high bit error rates (BER) after the conventional slice reconciliation procedure. This is because the continuous nature of the transmitted signals in Gaussian modulated CV-QKD means that valid signals can be arbitrarily close to the decision point chosen at the receiver. This contrasts with classical coherent systems, which transmit data in a well-defined constellation of allowable states. Low density parity check (LDPC) codes with low code rates are required to decode binary data blocks with high BERs. LDPC codes with low code rate (ratio of data to error correcting overhead) introduce high decoding computational overhead for error reconciliation. Therefore, to achieve real-time CV-QKD systems with high secret key rates, it is important to investigate methods to optimise slice reconciliation to reduce the BER of received data before error correction.

Here we demonstrate a practical, high-speed GMCS CV-QKD system with front-end optical hardware, real-time parameter monitoring and a back-end post processing toolchain that can distil secret keys. We also present a novel optimised slice reconciliation procedure which maximises secret key rates by multilevel slicing and introducing appropriate guard bands. A key principle of operation of this local local oscillator (LLO) system is the use of alternating signal and reference pulses within the CV-QKD system. This allows the optical phase of the signal pulse to be encoded via the phase difference between the reference and signal pulses. Thus Alice’s Local Oscillator (LO)a need not be transmitted to Bob, removing a potential attack method for an adversary^12, 18^. Alice and Bob’s LOs need not be locked exactly, as long as their frequency difference is small, with little phase drift occurring between reference and signal pulses.

## Experimental details

### CV-QKD hardware system

**Figure 1:**
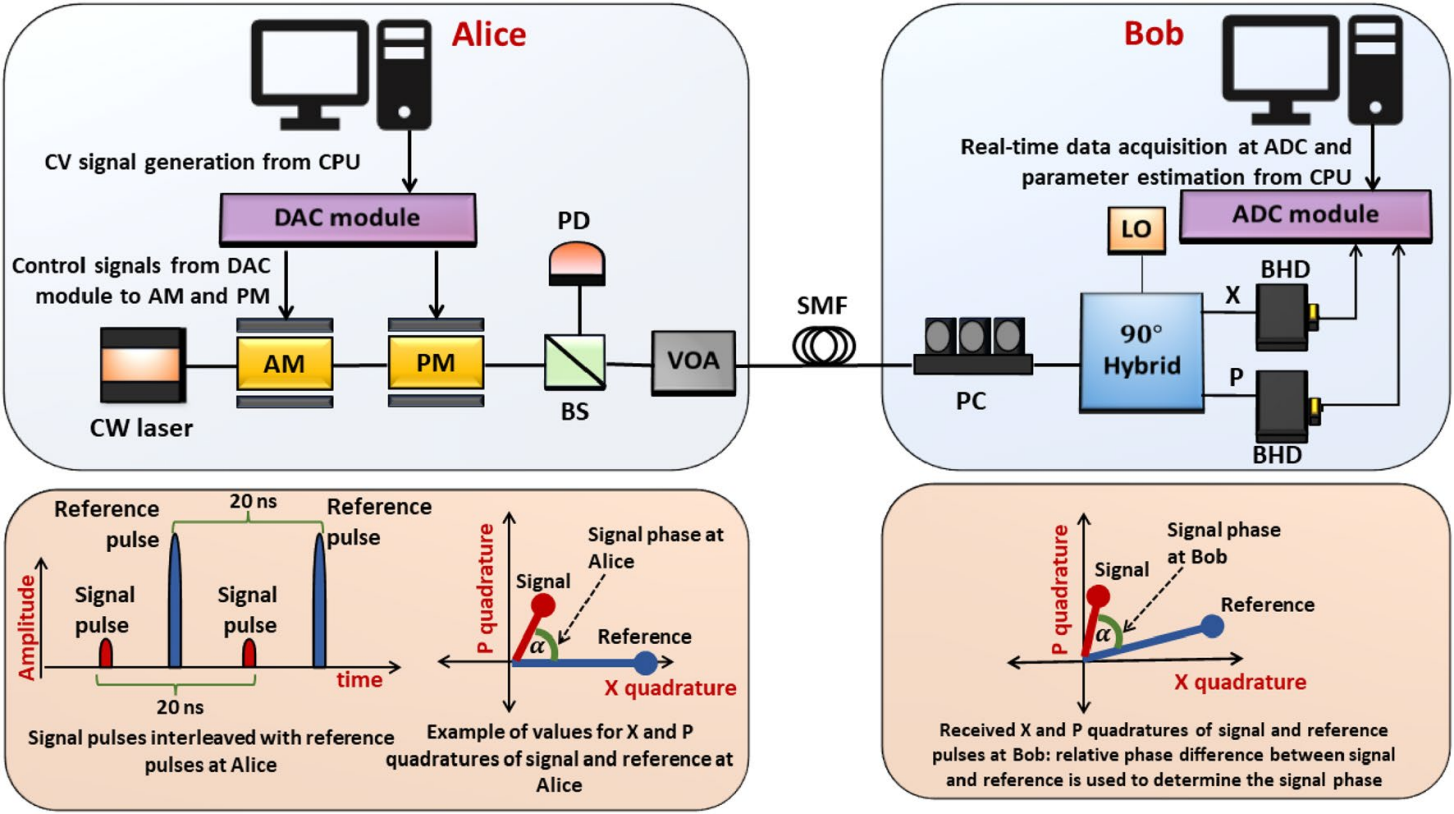
Optical hardware of our CV-QKD system: CW, continuous wavelength; AM, amplitude modulator; PM, phase modulator; BS, beam splitter; PD, photodiode; VOA, variable optical attenuator; SMF, single mode fibre; PC, polarisation controller; LO, local oscillator; BHD, balanced homodyne detector; X, X quadrature measured at Bob; P, P quadrature measured at Bob. An instance of X and P values of signal and reference pulses at Alice and Bob are also shown to highlight the relative phase between reference and signal pulse.

The hardware system implementation of our GMCS CV-QKD is shown in Fig. 1, with additional details of the components used being described in the “Methods” section. At Alice, a continuous wavelength, singlemode laser with a centre wavelength of 1550 nm and linewidth of 100 kHz is used. A 10 GHz bandwidth amplitude modulator with an extinction ratio of 22 dB applies intensity modulation to the continuous wavelength light to generate a train of interleaved signal and reference pulses at 100 MHz. Signal and reference pulses are interleaved in a manner that every signal pulse is followed by a reference pulse as shown in Fig. 1. Therefore, the resulting repetition rate of signal pulses is 50 MHz, and each optical pulse has a pulse width of 2 ns. A 10 GHz phase modulator introduces the required phases to the signal and reference pulses. These quantum signals are prepared with their quadratures following a zero centred Gaussian distribution with a specified variance $V_{A}$. The phase and amplitudes of the reference pulses are constant at Alice so that the arbitrary phase rotation of pulses observed at Bob can be compensated correctly. The control signals for the amplitude and phase modulators and synchronisation signals are generated by the high-speed DAC module integrated within Alice and Bob’s computers. The signal is split into two parts using a beam splitter, so that one output is used to monitor $V_{A}$ and the other is connected to a variable optical attenuator and the single mode fibre spool. In order to determine the average number of photons per pulse, we use a photodiode to measure the average optical power knowing the pulse repetition rate. For the sake of accuracy, the higher power reference pulses are absent during this measurement. The optical power is attenuated to the expected power level using the variable optical attenuator. We measured the average number of photons per signal pulse to be 60 $\pm$ 10.

At Bob, we use a derived local oscillator (LO) from the continuous wavelength laser at Alice and reference pulses to perform phase drift estimation and correction of quantum pulses. The path length of the optical fibre that transmits quantum signals and references is much longer than the length of the optical link of the LO arriving at Bob, resulting in the LO being incoherent with quantum and reference pulse^19^. Hence, phase noise introduced to the system is equivalent to having a second laser Bob with its wavelength aligned with the one at Alice. Received signals are passed through a polarisation controller which aligns them with the input polarisation of a 90-degree optical hybrid which combines them with the local oscillator. Both X and P quadratures of the coherent optical signal are then detected using two 400 MHz shot noise limited balanced homodyne detectors. The detected signals are then recorded in real time as digitised signals and transferred to the central processing unit (CPU) for post processing and real-time parameter monitoring by the high-speed ADC module located on Bob’s data acquisition board.

### Real-time parameter monitoring and post processing toolchain

**Figure 2:**
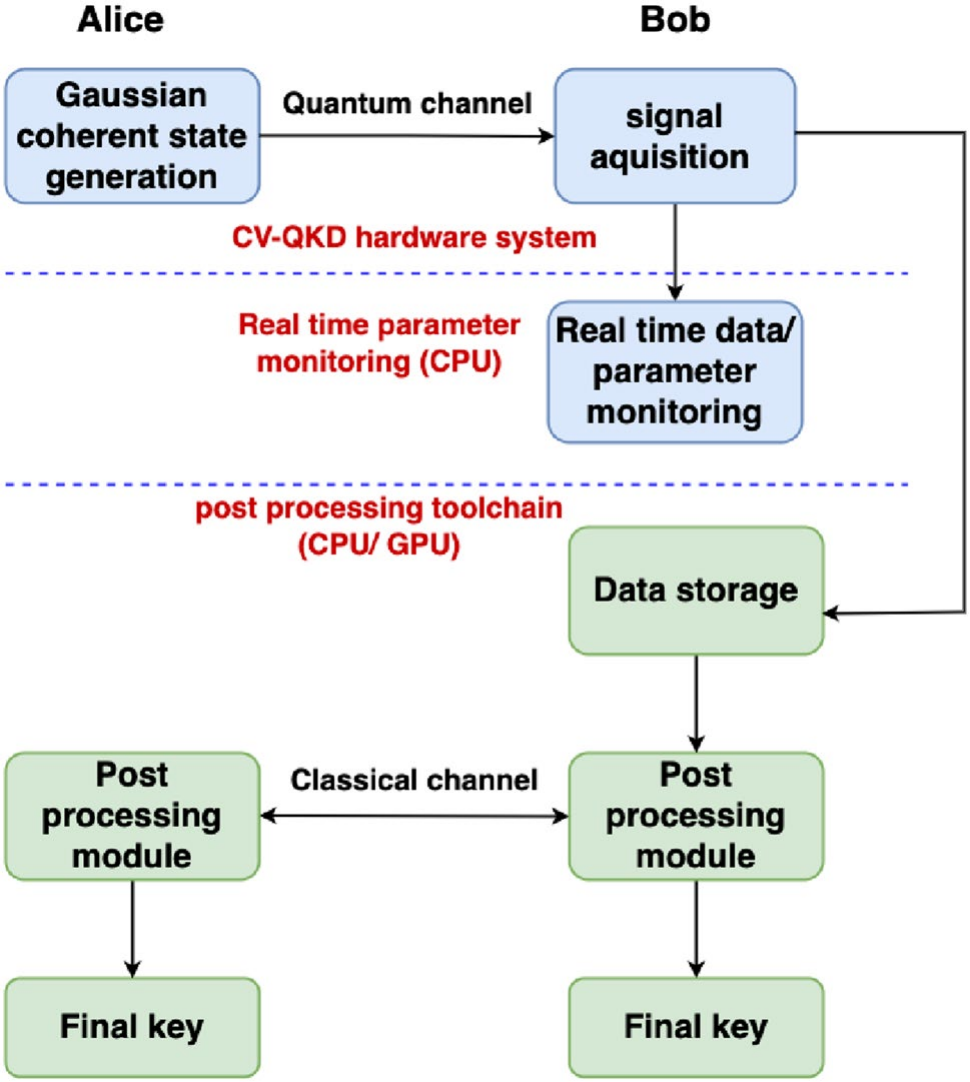
Schematic of the front-end optical hardware system, real-time parameter monitoring and back-end post processing toolchain of our GMCS CV-QKD system: Central processing unit (CPU), graphical processing unit (GPU).

The back-end real-time parameter monitoring software module and the post processing toolchain we have developed for our CV-QKD system are shown in the schematic diagram in Fig. 2. The software based real-time module performs two key functions. It first uses the analogue floating point data from the receiver to measure excess noise, shot noise and phase noise and enable phase drift compensation of the quantum signals using reference pulses. The purpose of the parameter monitoring is to detect and discard instances of potential unstable system operation in real-time and also to optimise the subsequent step of slice reconciliation. This slice reconciliation process, turns the floating point data in to a binary data stream, 10% of which is used to compare Alice and Bob’s raw bit error rate. The rest of the post-slicing data is then used for key distillation in the post processing toolchain.

Using the calculated BER, we apply a novel optimisation method for the slice reconciliation procedure to maximise the predicted secret key rate, this is described in detail in the next section. The parameters calculated from this optimisation are then communicated to the post processing toolchain in order to optimise the error correction procedure. The data from slice-reconciliation are then processed by error correction using LDPC with adaptive code rates chosen according to the raw BER. Finally, secret keys are distilled from a privacy reconciliation procedure using Toeplitz matrices. Currently, the post processing toolchain is run offline on a CPU. We are developing a real-time post processing toolchain based on graphical processing units (GPU), with coprocessors to support real-time privacy amplification^20^.

## Results

### Secret key and excess noise results

**Figure 3:**
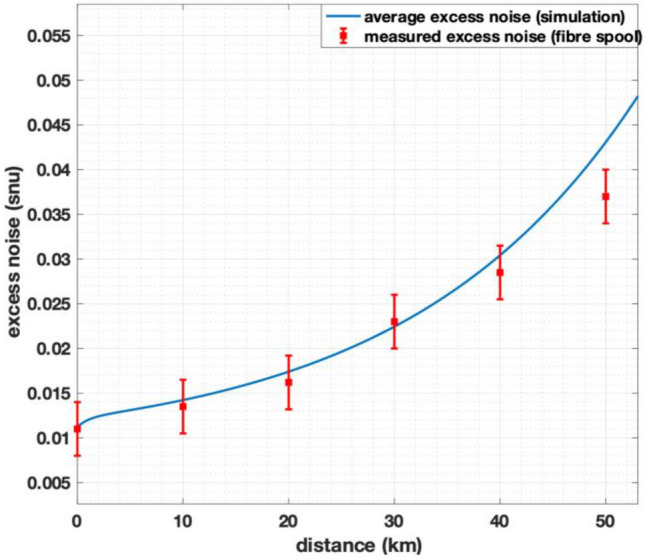
Excess noise measured from our real-time parameter monitoring system is shown by red markers with error bars. The blue curve shows the expected excess noise levels from our CV-QKD system using a simulation.

It is possible to predict secure key rates within the CV-QKD system based on excess noise values, but this assumes ideal slice reconciliation and error correction. Many reports of CV-QKD systems cite such excess noise values and the corresponding predicted secure key rates^11–13, 21^, which are seldom achievable in fully operational CV-QKD systems. In order to determine excess noise values within our system excess noise, data for each transmission distance are collected by the real-time monitoring software module over a period of several hours. These are then averaged. Results are presented in Fig. 3 together with predicted values obtained by numerical simulation of the system. Excess noise values are described in shot noise units (snu) and for instance, 0.05 ± 0.004 snu of excess noise is recorded at 20 km.

At Bob, we use a derived LO from the continuous wavelength laser at Alice and reference pulses to perform phase drift estimation of quantum pulses. As previously described, the phase noise introduced to the system is similar to having a local local oscillator (LLO) at Bob. Even the shortest transmission distance of 10 km is longer than the coherence distance of 1 km (for a 100 kHz linewidth source)^22, 23^; The additional phase noise introduced by this transmission is included in the simulation shown in Fig. 3. Figure 3 also shows excess noise measured from our system for different transmission distances of fibre.

**Figure 4:**
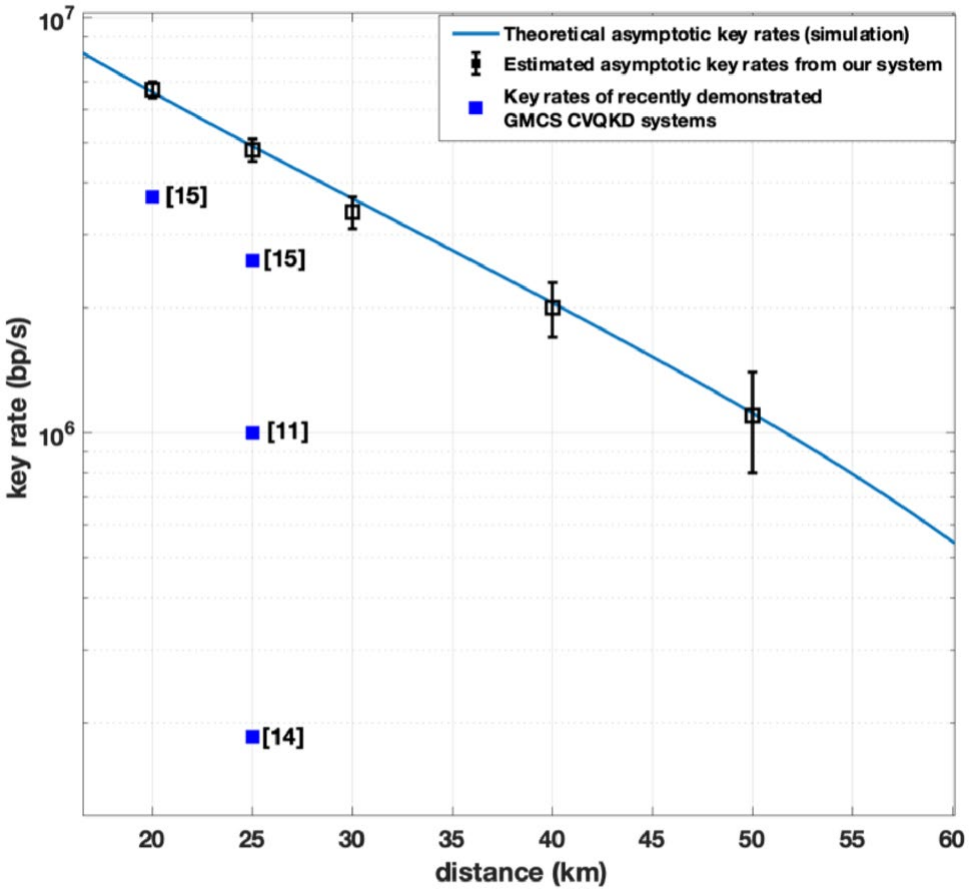
Asymptotic key rate vs. transmission distances for our CV-QKD system: the curve shows the asymptotic key rates from average noise measured from our system. Black points with error bars show the range of asymptotic key rates estimated from measured excess noise from the system. The blue points represent the key rate values from the corresponding references. Note that these asymptotic key rates assume infinite data block sizes and do not consider slicing with guard bands or privacy amplification.

For our system, asymptotic key rates of 6.2 Mb/s, 5.2 Mb/s 3.8 Mb/s, 1.8 Mb/s, and 0.9 Mb/s are estimated from measured average excess noise values for transmission distances of 20 km, 25 km, 30 km, 40 km, and 50 km respectively as shown by the black squares within Fig. 4. Note that these asymptotic key rates assume infinite data block sizes and do not consider slicing with guard bands or privacy amplification. In addition Fig. 4 also includes reports of key rates form other recent publications of GMCS CV-QKD demonstrations^11, 14, 15^.The theoretical asymptotic key rates estimated by a numerical simulation of our CV-QKD system are also shown in Fig. 4.

### Optimised slice reconciliation with guard bands

**Figure 5:**
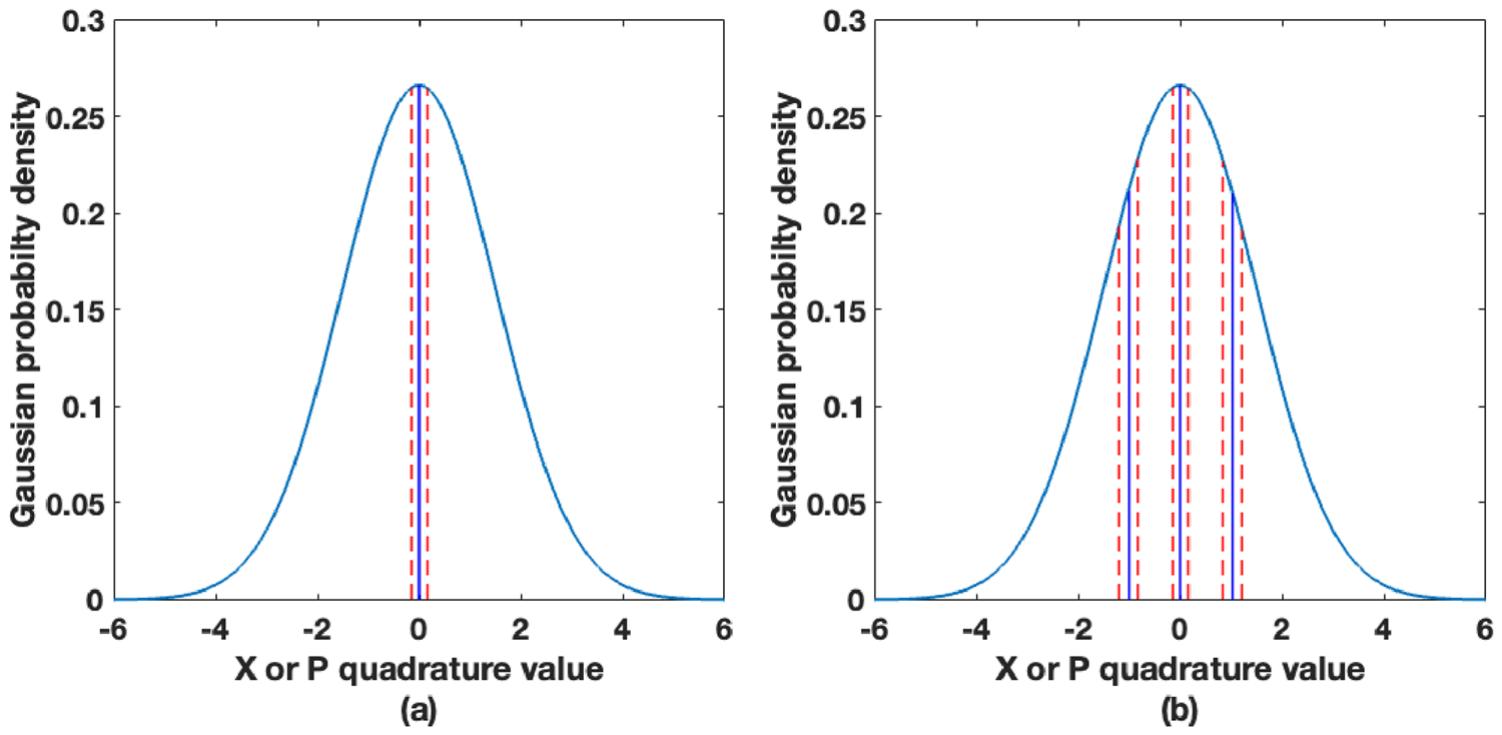
A diagram of guard bands in the zero centred Gaussian distributed continuous data for a variance of 2.12 when (**a**) m = 1 and (**b**) m = 2. X axis is the amplitude of continuous values and Y axis is the probability density. Blue lines show the slicing boundaries whilst the red dashed lines show guard bands.

The real-time process of slice reconciliation converts continuous variables at Alice and Bob into binary strings of data. To apply slice reconciliation, a set of $$m$$ slice functions $$S_{1} , \ldots ,S_{m} :{\mathbb{R}} \to \left\{ {0,1} \right\}$$ is chosen^16, 17^. These slicing functions transform a continuous variable source to an $$m$$ bit source and divide the real number line into $$2^{m}$$ disjoint intervals with $$2^{m}$$$$-1$$ slicing interval boundaries. The resulting $$m$$ bit sources at Alice and Bob after applying the slicing functions are corrected for bit errors using LDPC^17^. Here, we consider instances when $$m=1$$ with $$2$$ slicing intervals ($$1$$ slicing interval boundary around zero), and when $$m=2$$ with $$4$$ slicing intervals ($$3$$ slicing interval boundaries). The slicing intervals are assigned in such a way that the data is divided into equal probability intervals. Slicing of Gaussian distributed data when $$m=1$$ and $$m=2$$ is shown in Fig. 5.

If there is an sharp decision level at the slicing point, recorded data, which contain some uncertainty introduced by excess noise principally at the detector, may be assigned to the wrong slice, resulting in bit errors.

**Figure 6:**
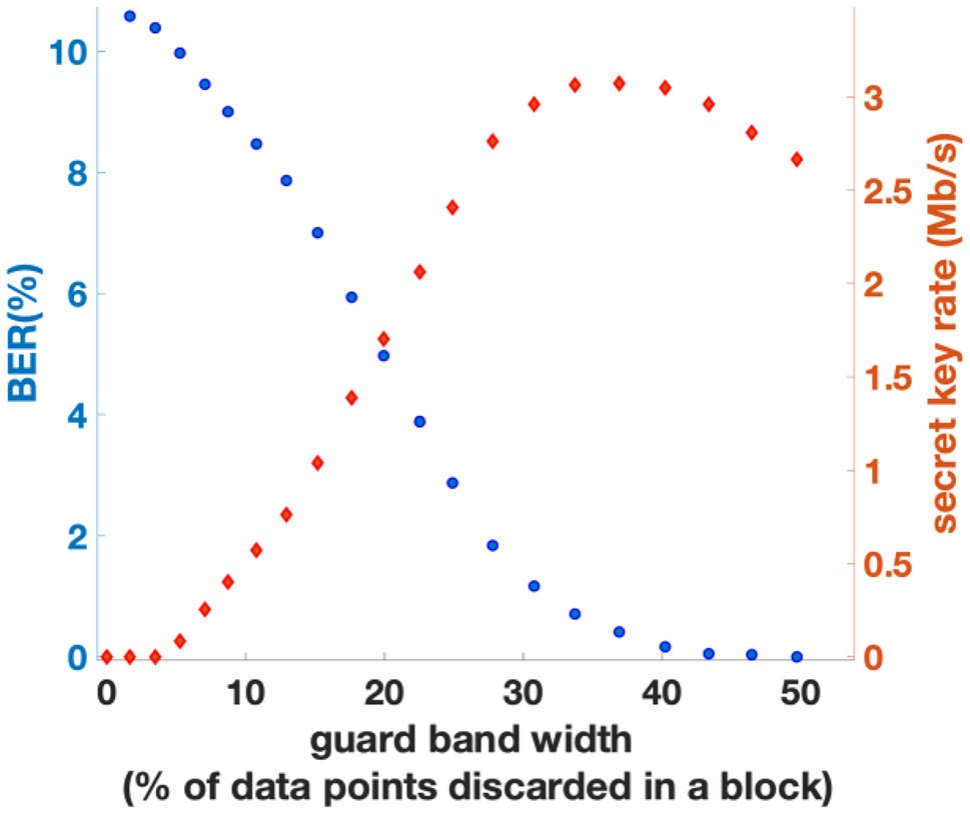
BERs and secret key rate with 1 guard band (m = 1) at a transmission distance of 25 km.

We have therefore introduced guard bands around slicing interval boundaries to decrease the BERs of the bit strings obtained after slice reconciliation. Since the amplitudes of quantum signals detected are low due to the low received SNR values, we have observed in our experimental data that without guard bands, a large number of bit errors occur near the slicing intervals. With high BERs, the decoding computational overhead for error correction using LDPC is significant since LDPC with low code rates are required. The guard bands around slicing thresholds reduce overall BER at the expense of some data being discarded from the original blocks of data received at Bob. After guard bands are applied at Bob, the indices of the discarded data points are communicated back to Alice via the classical communication channel, instructing Alice to discard those corresponding data points. We assume that communication of these indices to Alice over the classical channel leak a negligible amount of information to eavesdroppers. Security of multi-level slicing with guard bands is discussed further in “Methods” section. Guard bands are shown in Fig. 6 for one guard band $(m=1)$ and three guard bands $(m=2)$.

There is however a trade-off in the optimisation of guard band size. If too small a guard band is chosen the resulting increased BER will cause a reduction in throughput because of the reduction of the secure key size owing to aggressive privacy amplification. On the other hand, too large a guard band will result in excessive discarding of initial data points, resulting in reduced key material availability and hence reduced secret key rate. To optimise, we determine the widths of guard bands at each slicing interval boundary to decrease the BER without discarding too much data, so the secret key rate after all the post processing stages is maximised. To calculate the secret key rates, we firstly sample and compare 10% of Alice’s and Bob’s data in a data block to measure the BER in real time. With this BER value, the amount of privacy amplification required to distil secret keys is derived and the final secret key rates from our CV-QKD system can be predicted. The method for calculating the final secret key rates by considering post processing is given in the “Methods” section of this paper.

Figure 6 shows the resulting BER and secret key rates after 25 km transmission when one guard band centred around zero is applied $(m=1)$. As shown in Fig. 8, secret key rates of 3.8 Mb/s, 3.0 Mb/s, and 2.3 Mb/s were achieved for transmission distances of 20 km, 25 km, and 30 km respectively when $m=1$.

**Figure 7:**
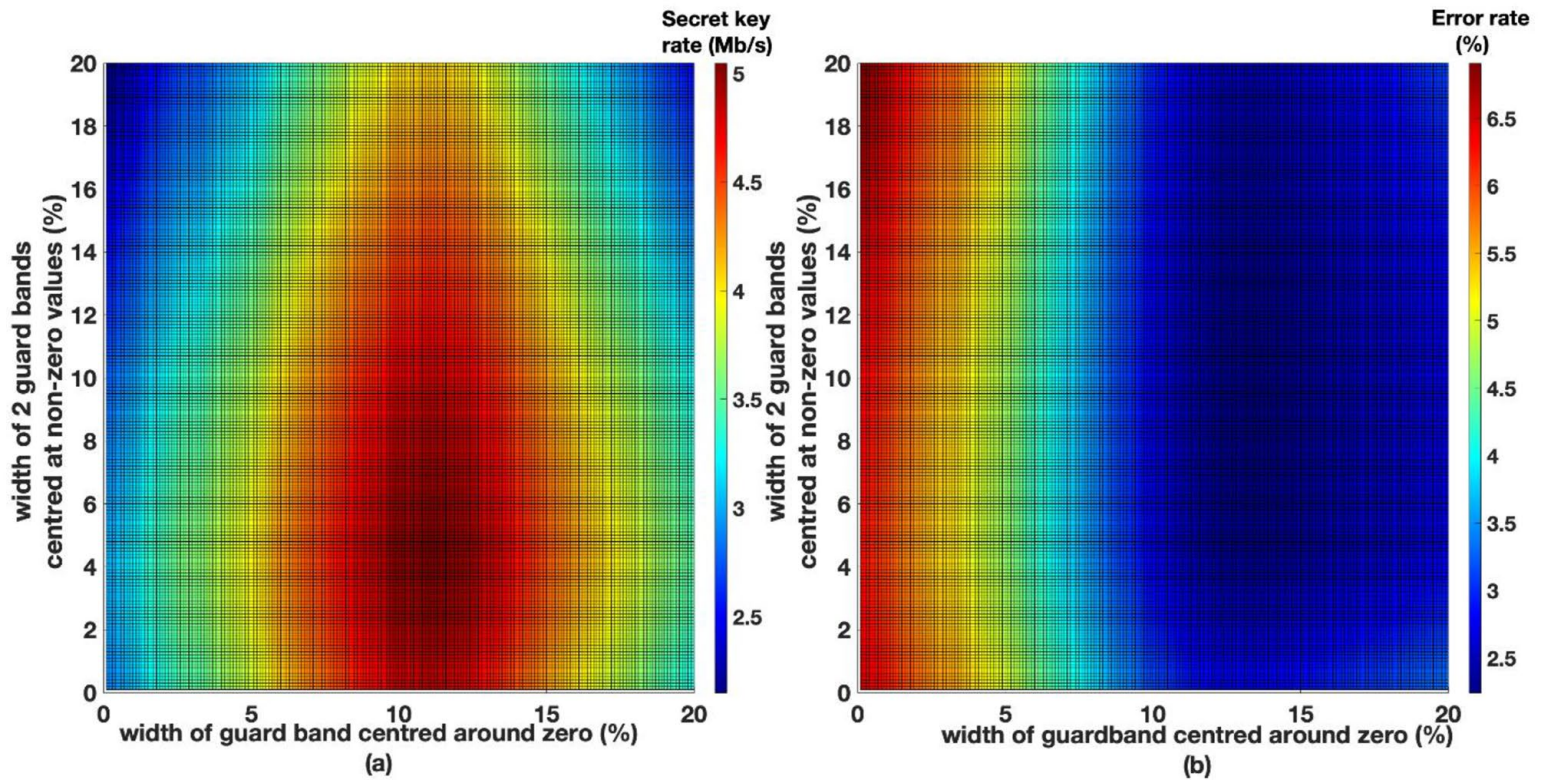
(**a**) Secret key rates with 3 guard bands (m = 2) at a transmission distance of 25 km. Guard band widths are shown as a percentage of data range of the Gaussian distribution of X or P quadratures. (**b**) BERs with 3 guard bands (m = 2) at a transmission distance of 25 km. Guard band widths are shown as a percentage of data range of the Gaussian distribution of X or P quadratures.

When $m=2$, three guard bands are needed, out of which, one is centred around zero and the other two are centred around arbitrary nonzero values of the Gaussian distribution of the quantum signals. Since the Gaussian distribution is symmetrical around zero, the two guard bands that are centred around nonzero values should have identical widths. The widths of these three guard bands are optimised to find the maximum secret key rate achievable, while decreasing the BER. The secret key rate calculation is carried out following the same process as described previously. At transmission distances of 20 km, 25 km, and 30 km, final secret rates of 6.2 Mb/s, 4.9 Mb/s, and 3.7 Mb/s were achieved when $m=2$. Secret key rates and BER attained when $m=2$, at transmission distances of 25 km shown in Fig. 7a and b respectively.

**Figure 8:**
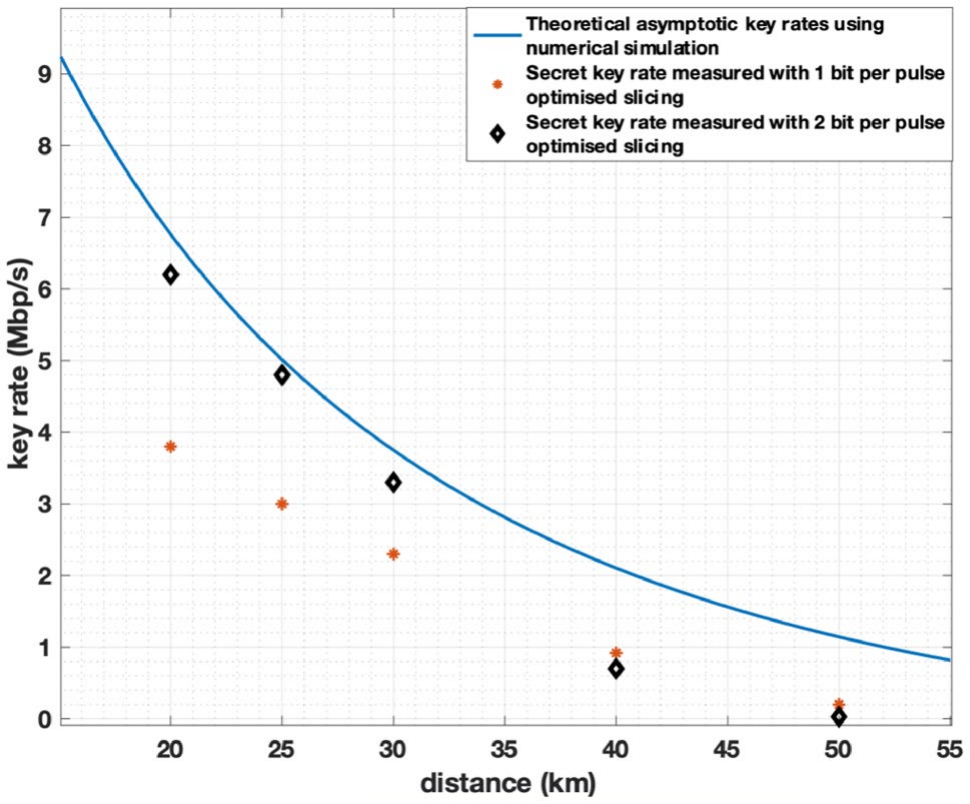
Theoretical asymptotic key rates calculated using numerical simulations (blue curve) compared with secret key rates obtained using 1 bit per pulse optimised slicing (red stars) and 2 bit per pulse optimised slicing (black diamonds).

As shown in Fig. 8, it can be observed that when moving from one slice to three slices, hence from 1 to 2 bits per symbol, the secret key rates can be increased from 2.4 to 4.7 Mb/s after a transmission distance of 30 km. Figure 8 shows that for moderate excess noise levels, with two level slicing, secret key rates that are close to theoretical key rate can be achieved. At higher transmission distances, when excess noise is very high, single level slicing performs better than multi-level slicing.

## Discussion

We have demonstrated a GMCS CV-QKD system with a symbol rate of 50 MHz, the highest achieved with a real-time front-end system. Our system consists of front-end optical hardware and a back-end software toolchain for monitoring system parameters in real-time and post processing to distil secret keys. Our software module can record received signals at Bob and calculate system parameters in real time. For one level slicing, 1 bit per symbol, we have produced a record 3.0 Mb/s final secret key rate after a transmission distance of 25 km for practical GMCS CV-QKD systems.

In addition, we have realised, for the first time, slice reconciliation for multilevel slicing with guard bands to decrease BER, thus maximising the final secret key rate. We have implemented the optimised slice reconciliation scheme in real time on our CV-QKD system. Secret key rates are increased from 2.3 to 3.7 Mb/s when moving from one slice $(m=1)$ to three slices $(m=2$), hence from 1 to 2 bits per symbol, at a transmission distance of 30 km. These are record secret key rates for real time, practical GMCS CV-QKD systems.

We are developing our post processing toolchain to be run on GPUs since currently, the post processing toolchain is executed on a CPU. It is expected that with our post processing toolchain run on GPUs will achieve secret key distillation in real time at the aforementioned secret key rates. Our analysis of optimisation of multilevel slicing was presented with experimental results in this paper. Therefore, we are currently expanding our work to include a theoretical approach for the optimised slice reconciliation procedure.

## Methods

### CV-QKD hardware system

At Alice, a continuous wavelength, singlemode laser (Thorlabs SFL 1550P) with a centre wavelength of 1550 nm and linewidth of 100 kHz is used. A 10 GHz amplitude modulator (iXblue MX-LN-10) converts the continuous wavelength light into 2 ns optical pulses at a repetition rate of 100 Hz and applies intensity modulation to create a train of interleaved signal and reference pulses. A 10 GHz phase modulator (iXblue MPZ-LN-10) sets the required phase of both signal and reference pulses. The control signals for amplitude and phase modulators and synchronisation signals are generated by the high-speed DAC module integrated within Alice and Bob’s computer (X6-1000M). To amplify the modulation signals from the DAC, current feedback amplifiers (Texas instruments THS3491) are used. The optical signal is split into two parts so that one output is used to monitor ${V}_{A}$ and the other is connected to a variable optical attenuator and the single mode fibre spool.

At Bob, X and P quadratures of the coherent optical signal are then detected using two 400 MHz shot noise limited balanced homodyne detectors (ThorLabs PDB470C). The detected signals are then recorded in real time as digitised signals and transferred to the CPU for post processing and real-time parameter monitoring by the high-speed ADC module located on Bob’s data acquisition board (X6-1000 M).

### CV-QKD parameter monitoring

The channel parameters that are measured in our real-time software module are transmittance $T$ and excess noise $\xi$. These channel parameters are estimated by Eqs. (1) and (2)^24, 25^:

1 $$V_{B} = \gamma \eta TV_{A} + N_{0} + \gamma \eta T\xi + V_{ele}$$

2 $$X_{A} X_{B} = \sqrt {\gamma \eta T} V_{A}$$

where $X_{A}$ and $X_{B}$ are the $X$ quadrature values at Alice and Bob respectively. These equations can be applied to P quadrature in the same manner. In these equations, $\eta = 0.5$ is the efficiency of the balanced homodyne detector at Bob and $V_{ele} = 0.1N_{0}$ is the electronic noise variance where $N_{0}$ is the shot noise variance. In our CV-QKD system, we monitor $N_{0}$ in real time. These parameters are independently estimated in our system. $\gamma = 1/2$ is for heterodyne detection, which is used in our system. $\gamma = 1$ if homodyne detection was used. Moreover, $V_{A}$ and $V_{B}$ are the variance of $X$ or $P$ quadratures at Alice and Bob respectively. We estimate these parameters from our system in real time with 10% of the data in a block of $10^{6}$ data points.

### CV-QKD secret key rate estimation

We perform secret key rate estimations under collective attacks for reverse reconciliation^24–26^.

Under collective attacks, asymptotic key rate $K$ can be given in (3), where $R$ is the effective symbol rate, $\beta$ is the reconciliation efficiency:

3 $$K = R\left( {\beta I_{AB} - \chi_{BE} } \right).$$

The mutual information between Alice and Bob $I_{AB}$ is given in (4):

4 $$I_{AB} = \log_{2} \frac{{V + \chi_{tot} }}{{1 + \chi_{tot} }}{. }$$

V and ${\upchi }_{{{\text{tot}}}}$ given in (4) are calculated as shown in Eqs. (5) and (6):

5 $$V = V_{A} + 1,$$

6 $$\chi_{tot} = \chi_{line} + {\raise0.7ex\hbox{${\chi_{het} }$} \!\mathord{\left/ {\vphantom {{\chi_{het} } T}}\right.\kern-0pt} \!\lower0.7ex\hbox{$T$}}.{ }$$

Channel noise ${\upchi }_{{{\text{line}}}}$ is given by Eq. (7):

7 $$\chi_{line} = \frac{1}{T} - 1 + \xi .$$

Heterodyne detection ${ }\chi_{het}$ noise is given by Eq. (8):

8 $$\chi_{het} = \left( {2 + 2V_{ele} - \eta } \right)\eta .{ }$$

As shown in Eq. (3), the key rate under collective attack is bound by the Holevo bound ${\chi }_{BE}$ which is the maximum information which can be gained by Eve under collective attack, as given in Eq. (9).

9 $$\chi_{BE} = \mathop \sum \limits_{i = 1}^{2} G\left( {\frac{{\lambda_{i} - 1}}{2}} \right) - \mathop \sum \limits_{i = 3}^{5} G\left( {\frac{{\lambda_{i} - 1}}{2}} \right),$$

where ${\text{G}}\left( {\text{x}} \right)$ is given by Eq. (10)

10 $$G\left( x \right) = \left( {x + 1} \right)\log_{2} \left( {x + 1} \right) - x\log_{2} x$$

The eigenvalues ${\lambda }_{i}$ given in Eq. (9) can be evaluated as shown in Eqs. (11), (12) and (13):

11 $${\uplambda }_{1,2} { } = \sqrt {\frac{1}{2}\left( {{\text{A}} \pm \sqrt {{\text{A}}^{2} - 4{\text{B}}} } \right)} ,$$

12 $${\uplambda }_{3,4} = \sqrt {\frac{1}{2}\left( {{\text{C}} \pm \sqrt {{\text{C}}^{2} - 4{\text{D}}} } \right)} ,{ }$$

13 $${\uplambda }_{5} { } = 1.{ }$$

The terms A, B, C, and D in Eqs. (11–13) are evaluated as shown in Eqs. (14–17):

14 $${\text{A }} = {\text{V}}^{2} \left( {1 - 2{\text{T}}} \right) + 2{\text{T}} + {\text{T}}^{2} \left( {{\text{V}} + {\upchi }_{{{\text{line}}}} } \right)^{2} ,$$

15 $${\text{B }}\; = \;\left[ {{\text{T}}\left( {{\text{V}}\chi_{{{\text{line}}}} + 1} \right)} \right]^{2} ,$$

16 $${\text{C}}\; = \;\frac{{\left[ {{{{\rm A} \chi }}_{{{\text{het}}}}^{2} + {\text{B}} + 1 + 2{\upchi }_{{{\text{het}}}} \left( {{\text{V}}\sqrt {\text{B}} + {\text{T}}\left( {{\text{V}} + {\upchi }_{{{\text{line}}}} } \right)} \right) + 2{\text{T}}\left( {{\text{V}}^{2} - 1} \right)} \right]}}{{\left( {{\text{T}}\left( {{\text{V}} + {\upchi }_{{{\text{tot}}}} } \right)} \right)^{2} }}$$

17 $${\text{D }}\; = \;\left[ {\sqrt {\text{B}} \left( {{\text{V}} + \sqrt {\text{B}} {\upchi }_{{{\text{het}}}} } \right)/{\text{T}}\left( {{\text{V}} + {\upchi }_{{{\text{tot}}}} } \right)} \right]^{2} .$$

In order to calculate the final secret key rates, the key length $\mathrm{l}$ that can be extracted from a universal hash function must be determined. To determine the amount of privacy amplification required BER is also calculated using 10% of the data points a block. This BER is monitored in real time so that the amount of privacy amplification required to distil secret keys can be determined in real time. Maximum key length after privacy amplification ${\text{l}}_{{{\text{max}}}}$ after slice reconciliation procedure with bit strings present at both Alice and Bob can be determined as given in Eq. (18):

18 $${\text{l}}_{{{\text{max}}}} = {\text{n}}\left( {1 - {\text{h}}\left( {{\text{Q}}_{{{\text{tol}}}} } \right)} \right) - {\text{leak}}_{{{\text{EC}}}} .$$

where n is the amount of data points used in a data block for key distillation (in our case, we use 90% of data in ${10}^{6}$ data blocks), h is the binary entropy function, ${\mathrm{Q}}_{\mathrm{tol}}$ is the channel error tolerance and ${\mathrm{leak}}_{\mathrm{EC}}$ is the amount of information which need to be exchanged by Alice and Bob during error reconciliation stage as detailed in^27, 28^.

### Security of multi-level slicing with guard bands

In our CV-QKD system, quantum signal transmission and reception follow the standard Gausssian modulated coherent state (GMCS) CV-QKD protocol. Multi-level silicing procedure we use, in order to convert the continuous variables in to a bit stream is a standard procedure for GMCS CV-QKD. Therefore, the security of multi-level slicing procedure is shown in previous work^16, 17^.

We apply guard bands to our data as a post processing step, after the standard quantum signal transmission steps of GMCS CV-QKD. The slicing thresholds for X and P quadrature distributions are chosen arbitrarily by Bob and we assume that Bob’s unit is secure from an adversary. In order to communicate which data points that Alice should discard, Bob sends the time stamps of data points that lie within the guard bands (indices of data points in each data block that lie within the guard band) over the classical communication channel. Since we do not communicate any information related to the thresholds of guard bands or the actual X and P values of the quantum signals received at Bob, we assume that the amount of additional information which is leaked to an adversary is negligible when guard bands are applied at the post-processing stage. Since we follow the standard GMCS CV-QKD at the quantum communication stage, we can compare the security of our GMCS CV-QKD system to the standard security model of GMCS CV-QKD.

To justify the security of guard bands, we consider it as a form of non-Gaussian post-selection procedure since discarding data points that lie within the guard bands alter the original Gaussian distribution of data sent from Alice. Our guard band post-selection closely resembles non-Gaussain post-selection used in discrete modulated CV-QKD protocols^29, 30^. However, our post-selection procedure is unique in that it is optimised specifically to improve the performance of slice reconciliation.

## Data Availability

Additional data related to this publication is available at 10.17863/CAM.104101.
